# Competing paradigms of obesity pathogenesis: energy balance versus carbohydrate-insulin models

**DOI:** 10.1038/s41430-022-01179-2

**Published:** 2022-07-28

**Authors:** David S. Ludwig, Caroline M. Apovian, Louis J. Aronne, Arne Astrup, Lewis C. Cantley, Cara B. Ebbeling, Steven B. Heymsfield, James D. Johnson, Janet C. King, Ronald M. Krauss, Gary Taubes, Jeff S. Volek, Eric C. Westman, Walter C. Willett, William S. Yancy, Mark I. Friedman

**Affiliations:** 1 New Balance Foundation Obesity Prevention Center, Boston Children’s Hospital, Boston, MA USA; 2 Department of Pediatrics, Harvard Medical School, Boston, MA USA; 3 Department of Nutrition, Harvard T.H. Chan School of Public Health, Boston, MA USA; 4 Division of Endocrinology, Diabetes and Hypertension, Brigham and Women’s Hospital, Harvard Medical School, Boston, MA USA; 5 Department Comprehensive Weight Control Center, Weill Cornell Medicine, New York, NY USA; 6 Obesity and Nutrition Science, the Novo Nordisk Foundation, Hellerup, Denmark; 7 Department of Medicine, Weill Cornell Medicine, New York, NY USA; 8 Metabolism & Body Composition Laboratory, Pennington Biomedical Research Center, Baton Rouge, LA USA; 9 Life Sciences Institute, University of British Columbia, Vancouver, BC Canada; 10 Department of Nutritional Sciences & Toxicology, UC Berkeley, Berkeley, CA USA; 11 Departments of Pediatrics and Medicine, UC San Francisco, San Francisco, CA USA; 12 Independent journalist, Oakland, CA USA; 13 Department of Human Sciences, Ohio State University, Columbus, OH USA; 14 Department of Medicine, Duke University School of Medicine, Durham, NC USA; 15 Monell Chemical Senses Center, Philadelphia, PA USA

**Keywords:** Obesity, Pathogenesis

## Abstract

The obesity pandemic continues unabated despite a persistent public health campaign to decrease energy intake (“eat less”) and increase energy expenditure (“move more”). One explanation for this failure is that the current approach, based on the notion of energy balance, has not been adequately embraced by the public. Another possibility is that this approach rests on an erroneous paradigm. A new formulation of the energy balance model (EBM), like prior versions, considers overeating (energy intake > expenditure) the primary cause of obesity, incorporating an emphasis on “complex endocrine, metabolic, and nervous system signals” that control food intake below conscious level. This model attributes rising obesity prevalence to inexpensive, convenient, energy-dense, “ultra-processed” foods high in fat and sugar. An alternative view, the carbohydrate-insulin model (CIM), proposes that hormonal responses to highly processed carbohydrates shift energy partitioning toward deposition in adipose tissue, leaving fewer calories available for the body’s metabolic needs. Thus, increasing adiposity causes overeating to compensate for the sequestered calories. Here, we highlight robust contrasts in how the EBM and CIM view obesity pathophysiology and consider deficiencies in the EBM that impede paradigm testing and refinement. Rectifying these deficiencies should assume priority, as a constructive paradigm clash is needed to resolve long-standing scientific controversies and inform the design of new models to guide prevention and treatment. Nevertheless, public health action need not await resolution of this debate, as both models target processed carbohydrates as major drivers of obesity.

“[P]roponents of competing paradigms practice their trades in different worlds … Both are looking at the world, and what they look at has not changed. But in some areas they see different things, and they see them in different relations one to the other. That is why a law that cannot even be demonstrated to one group of scientists may occasionally seem intuitively obvious to another.” – T. Kuhn, 1970 [1].

Textbooks, public health guidelines and patient education materials characteristically conceptualize obesity as a disorder of energy balance. A scientific statement from the Endocrine Society concludes that “Obesity pathogenesis involves … sustained positive energy balance (energy intake > energy expenditure)” [2] and an expert panel report from major professional health associations asserts, “To achieve weight loss, an energy deficit is required” [3]. However, these reiterations of the first law of thermodynamics conflate physics with pathophysiology [4–7]. A gain in body energy stores—fat mass, for practical purposes—necessarily constitutes a positive energy balance; explaining the former by the latter is tautological. Clearly, fever can only develop in the presence of a positive “heat balance,” but patients with fever don’t require instruction in this self-evident concept and academic reviews of febrile illness don’t dwell on the physics of heat. Any useful biological hypothesis of obesity pathogenesis must consider causal direction, and the law of energy conservation allows more than one possibility.

According to the conventional view as reflected in the energy balance model (EBM), overeating drives excess adiposity. Dietary treatment focuses on decreasing energy intake to reduce body fat stores. An alternative view, the carbohydrate-insulin model (CIM), posits an opposite pathway—that increasing adiposity drives overeating. Dietary treatment aims instead to reduce body fat storage primarily through hormonal mechanisms that directly impact adipose tissue, thereby producing a negative energy balance. Figure 1 depicts this foundational contrast in mechanisms. (The terms “overeating” and “positive energy balance” are used interchangeably to mean energy intake > energy expenditure. Because adipose tissue is the body’s primary energy storage depot, increasing body fat mass indicates the presence of a positive energy balance, and vice versa).

**Fig. 1:**
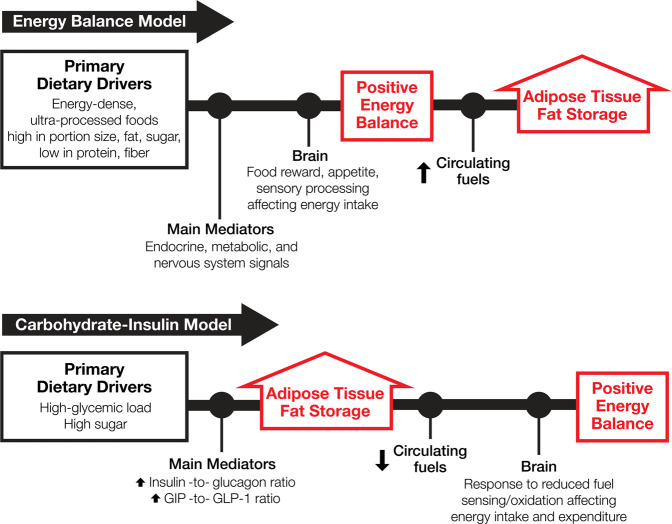
Contrasting causal pathways in obesity models. The first law of thermodynamics dictates that a positive energy balance must exist as body energy stores increase. *Positive Energy Balance* is upstream of increased *Adipose Tissue Fat Storage* in the Energy Balance Model [9] and downstream in the Carbohydrate-Insulin Model [8]. (These representations are not intended to include all mediating or modifying environmental and pathophysiological influences.).

Although versions of these two models have competed for almost a century, this controversy recently intensified, as highlighted by expanded formulations of the CIM by Ludwig et al. [8] and EBM by Hall et al. [9] in *The American Journal of Clinical Nutrition*. The aim of this review is to compare features of both models, assess strengths of the supporting evidence, and specify improvements in formulation of the EBM to promote a constructive paradigm clash.

## The new energy balance model—a focus on food intake

Both models of obesity share a common feature: presumed homeostatic regulation of a critical physiological parameter to promote optimal functioning [10, 11]. In the EBM, body weight (or body fat) is the regulated variable, a possibility with some evolutionary support: whereas adequate body fat is needed for survival during times of food scarcity, excessive fat might increase risk of predation.

The new EBM of Hall et al. [9] proposes that the brain controls food intake to regulate body weight through “complex endocrine, metabolic, and nervous system signals acting in response to the body’s dynamic energy needs as well as environmental influences.” This control system centers on “reward, appetite, [and] sensory processing” involving “salience, wanting, and motivation that primarily operate below our conscious awareness.” Obesity results from “increased availability and marketing of a wide variety of inexpensive, convenient, energy-dense, ultra-processed foods that are high in portion size, fat, and sugar, and low in protein and fiber.” These exposures cause overeating, with the energy excess deposited into body fat.

Earlier formulations characteristically considered both components of energy balance in concert [12–17]. The EBM of Hall et al. [9] differs from these formulations, with a primary focus on the control of food intake and less attention to energy expenditure. This new EBM implies, and related reviews explicitly state [2, 18–20], that all calories are metabolically alike in the model. For instance, Hall and Guo [19] assert that, “for all practical purposes, ‘a calorie is a calorie’ when it comes to body fat and energy expenditure differences between controlled isocaloric diets varying in the ratio of carbohydrate to fat.” While acknowledging that dietary composition influences oxidation rates of respective macronutrients, the EBM holds that diet ultimately drives fat deposition by increasing total energy intake, not through calorie-independent effects on substrate partitioning.

## The carbohydrate-insulin model—a special case of the metabolic paradigm

The CIM represents an opposing paradigm, with origins in the early twentieth century [7, 21–28], that considers the supply of metabolic fuels in the blood (as proxy for fuel oxidation) the regulated parameter. Whereas adequate body fat may aid survival during famine, access to metabolic fuels is required for immediate survival, in view of the dependency of all tissues, and especially the brain, on a continuous fuel supply [28–30].

The CIM [8, 31, 32] proposes that a high-glycemic load (GL) diet—one with large amounts of rapidly digestible carbohydrates (i.e., free sugar, processed grains, most starchy vegetables)—elicits hormonal responses that inhibit fat mobilization (lipolysis) and promote fat deposition in adipose tissue. As recently detailed [8], consumption of a high-GL meal produces a high ratio of insulin to glucagon secretion, and of GIP to GLP-1 secretion. This highly anabolic hormonal profile shifts substrate partitioning toward deposition, leaving less energy available for metabolically active tissue including the brain, especially in the late postprandial period [33, 34]. The brain responds to this metabolic state by activating pathways controlling hunger and other appetitive responses [35, 36] to promote energy intake. If an individual resists the drive to eat by restricting food, metabolic fuels are conserved through reduced energy expenditure manifesting as fatigue (leading to sedentary behavior), decreased non-exercise activity thermogenesis, increased muscular efficiency, and other mechanisms. Without a degree of calorie restriction beyond most people’s ability to sustain, fat accumulation results because of continued partitioning of energy into adipose tissue. Thus, the CIM offers an explanation for the poor efficacy of calorie-restricted diets beyond lack of adherence due to hedonic and reward influences.

In addition to GL, the CIM provides a conceptual framework for understanding how other dietary factors, behaviors and environmental exposures may affect body weight through metabolic mechanisms rather than primary effects on energy intake or expenditure; these include fructose [37–40], protein amount [41], fatty acid type, fiber, food order within a meal [42], meal timing [43], physical activity and endocrine-disrupting food additives and pollutants [44, 45]. The CIM also postulates a diet-phenotype interaction, such that individuals with high endogenous insulin secretion, disorders in glucose homeostasis, and high sensitivity to insulin-mediated suppression of adipocyte lipolysis would be especially susceptible to the adverse metabolic effects of a high-GL diet, potentially explaining some of the marked heterogeneity in response to macronutrient-focused weight loss diets [46–48].

This view of pathophysiology accords with the development of common forms of obesity. A small shift of substrate partitioning favoring fat storage would account for slow but progressive weight gain, until adipose tissue insulin resistance develops to a sufficient degree. Adipose tissue insulin resistance would counterbalance the excessive insulin secretion of a high-GL diet, resulting in a weight plateau, but at the cost of ectopic lipid deposition and systemic metabolic dysfunction, consistent with the adipose tissue expandability hypothesis [49]. This expanded formulation provides detailed mechanisms and numerous testable hypotheses to inform research [8].

## Evidence pertaining to the two models

The natural course of obesity, which usually develops over years to decades, involves excessive storage of ∼1 to 2 g fat/d on average—far too small to measure in short-term metabolic feeding studies (i.e., ≤2 weeks). Whereas this effect could be observable in longer-term outpatient trials and observational studies, causal inference from these data may be limited by poor adherence to test diets and confounding. Furthermore, few studies have focused on childhood, a dynamic stage of obesity development [50]. Although animal studies can elucidate mechanisms, their translation to humans remains problematic. For these reasons, the vast literature on obesity pathogenesis can be selectively cited to make opposing points, as each side of this debate has claimed of the other.

In this section, we do not aim to provide a comprehensive review of the literature, but rather highlight main disagreements with Hall et al. [9], considering study design limitations. Table 1 summarizes key features distinguishing the models to facilitate this assessment. Prior reviews offer a range of perspectives for [12–16, 51–58] and against [6, 31, 32, 59–67] earlier versions of the EBM.

**Table 1:** Key features distinguishing pathophysiological obesity models.

Distinguishing features	Energy balance model	Carbohydrate-insulin model
Causal direction	Positive energy balance drives fat deposition	Fat deposition drives positive energy balance
Regulated variable	Variously: food intake, energy balance, body weight or fat mass	Metabolic fuel oxidation rate in critical organs^a^
Primary dietary drivers of pandemic	Variously: high fat intake; high energy-dense, highly palatable foods; cheap, convenient ultra-processed foods; high sugar, fat, salt with low protein, fiber	High-glycemic load carbohydrates, fructose
Key pathophysiological mechanisms	“complex endocrine, metabolic, and nervous system signals [that] control food intake”	Hormonal responses to food, especially the ratios of insulin to glucagon and GIP to GLP-1
Calorie-independent effects of diet on energy expenditure	No	Yes
Calorie-independent effects of diet on substrate partitioning or fat deposition	No	Yes
Reduced circulating metabolic fuels in late postprandial period on high- vs. low- GL diet	No	Yes
Effect modification by insulin secretion^b^	Not specified	Yes

The energy balance model and carbohydrate-insulin model both recognize complex, multi-factorial influences on body weight related to genetics, behavior, and environment. These distinguishing features provide a basis for comparing model validity in hypothesis-driven research. Note that the models are not necessarily mutually exclusive; evidence in support of both models may be found in different forms of obesity and under differing experimental conditions.

^a^Metabolic fuel concentration in the blood is a proxy for oxidation rate in critical organs (brain, liver). Metabolic fuel concentration generally reflects oxidation rate during the dynamic phase of obesity development; these may be dissociated during the compensatory phase, with development of insulin resistance.

^b^Individuals with high- vs low-insulin secretion hypothesized to have more adverse responses to a high-glycemic load diet.

### Animal research

Although rodents and humans have not evolved to eat the same diets, experimental animal research has been considered in this debate. Hall et al. [9] present as evidence against the CIM the observation that a diet of 70% carbohydrate and 10% fat protects rodents from obesity and one with 20% carbohydrate and 60% fat produces the most weight gain in some experimental conditions. Similarly, a recent study with 5 mouse strains concluded that increasing dietary fat, but not carbohydrate or protein, was associated with greater variations in food intake and body weight [68]. However, Tordoff and Ellis [69] found that rodent diets with equal amounts (by energy) of carbohydrate and fat were most obesogenic and deviations in either direction reduced weight gain. Adding to this heterogeneity, Kennedy et al. [70] concluded that a very-low-carbohydrate diet (with lower protein content) in mice “induces a unique metabolic state congruous with weight loss”. Clearly, this research must be extrapolated to humans with caution, in view of well described limitations involving idiosyncrasies of inbred strains, confounding from uncontrolled dietary exposures and dissimilar nutrition requirements of rodents and humans [71–74]. For instance, saturated fat and sugar often comprise most calories on high-fat rodent diets, a combination that causes hypothalamic inflammation and systemic insulin resistance [75–82].

These methodological issues can be avoided by direct examination of causal direction. Whereas hormonal responses to macronutrients may differ among species due to evolutionarily divergence, biological mechanisms affecting fat storage are highly conserved, enhancing potential translation of rodent studies to humans [83–85]. In the EBM, diet drives fat deposition by increasing food consumption. Therefore, when animals on an obesogenic diet are pair-fed to littermates on an isocaloric control diet, ensuring the same energy intake, effects on body composition should be identical.

This prediction often fails. Petro et al. [86] pair-fed mice 58% vs 11% fat diets for 11 weeks and observed greater adiposity in the high-fat group (24.1 vs 18.5%, *P* < 0.001), consistent with other findings [87–89]. Similar calorie-independent effects have been observed with high-sugar diets [90–93]. Although one could challenge the implications of these data by arguing rodents are more susceptible to such metabolic effects, that argument would undermine the validity of rodent macronutrient studies for understanding human obesity in the first place.

Studies of glycemic index (GI) offer another way to circumvent species-specific differences in macronutrient metabolism. In a line of investigation involving several rodent strains and species, the effects of GI were examined by substitution of starch type, controlling for macronutrients, saturated fat, sugar, and micronutrients [82, 94–97]. These studies demonstrate the following changes among animals consuming high- vs. low-GI diets, in this sequence: hyperinsulinemia, a shift in substrate partitioning favoring fat deposition, decreased energy expenditure, increased adiposity and weight gain – all prior to an increase in energy intake. When energy intake was restricted to prevent weight gain, the high-GI group still developed abnormal body composition. Despite consuming fewer calories, these animals had more body fat at the expense of lean body tissues [96]. Although multiple mechanisms (e.g., gut microbiome), may mediate these effects, they contradict a fundamental premise of the EBM, that diet composition has no calorie-independent effects on fat deposition.

Finally, Hall et al. [9] dismiss studies of insulin action as non-discriminating, but these provide another opportunity to test model predictions head-to-head. In the CIM, greater insulin secretion promotes fat storage through direct peripheral mechanisms [8]. The EBM, with its focus on the central actions of hormones, seems to predict the opposite, in view of the anorectic actions of insulin in the brain [98–102]. These studies of adiposity, involving chronic insulin administration and genetic models of reduced insulin secretion, support the CIM [103–109]. Downplaying the significance of these findings—that the peripheral calorie-independent actions dominate central calorie-dependent ones—risks creating an EBM so general as to be untestable, especially as Hall et al. [9] interpret the central anorectic effects of insulin following nasal injection in humans as evidence against the CIM.

### Brain and genetics

While “nervous systems have evolved to control energy intake,” as Hall et al. [9] state, the brain also controls virtually all aspects of metabolism [110–113], including glucose metabolism, as famously described by Claude Bernard in the 1850s [114]. Indeed, effects of dietary composition on body composition consistent with the CIM manifest commonly among animal models of obesity, as exemplified in Table 2. With restriction of energy intake to levels at or below that of controls, increased adiposity has been observed in experimental models affecting numerous brain pathways thought to mediate food intake, demonstrating the existence of peripheral metabolic actions of putative “hunger” or “satiety” hormones. In some of these models, excessive adiposity spontaneously develops without increased food intake or body weight. These findings seem at odds with a common interpretation of human genetic studies that attributes the greater prevalence of obesity-related polymorphisms in the brain vs. adipocyte as evidence for the EBM.

**Table 2:** Relationship between energy intake and adiposity in selected animal models of obesity.

Animal Model	↑ Adiposity without (or Before) ↑ weight	↑ Adiposity without (or Before) ↑ energy intake	↑ Adiposity with control of energy intake^a^
*Indicates causal direction in CIM*			
High-glycemic index diet [82, 94–97]	+		
MC3 receptor deficiency [206–208]	+		
*Suggests causal direction in CIM*			
AgRP neuron ablation (regular chow) [209]		+	
CHOP deficiency [210, 211]		+	
GABA deficiency [212]		+	
High-sugar (fructose) diet^b^ [90–93]		+	
Monosodium glutamate brain lesion [213]		+	
MRAP2 deficiency [214]		+	
Ventromedial hypothalamic damage^b^ [25, 215, 216]		+	
*Consistent with causal direction in CIM*			
AgRP excess [217, 218]			+
Bombesin receptor subtype-3 deficiency [219]			+
High-fat diet [86–89]			+
Insulin excess [104, 107]			+
Leptin deficiency [220–222]			+
MCH excess^c^ [223]			+
MC4 receptor deficiency [224, 225]			+
Neuropeptide Y excess [226–228]			+
Opioid receptor-like 1 stimulation [229]			+

Increased adiposity before weight increase indicates causal direction in the CIM and contradicts that in the EBM. Increased adiposity without (or before) increased energy intake suggests causal direction in the CIM and tends to oppose that in the EBM. Increased adiposity with control of energy intake is consistent with causal direction in the CIM but does not exclude that in the EBM. (N.B., Pair-feeding or other controls to assess for the presence of a primary metabolic defect have not been conducted in many experimental animal models.) Although not reviewed here, animal models of leanness, such as genetic insulin knock down, demonstrate metabolic effects consistent with the CIM [108, 109]

*CHOP* CCAAT/enhancer-binding protein (C/EBP) homologous protein, *MCH* melanin concentration hormone, *MC3* melanocortin-3, *MC4* melanocortin-4, *MRAP2* melanocortin 2 receptor accessory protein 2.

^a^An increase in energy intake observed before an increase in adiposity does not exclude the CIM-specified causal pathway, due to imprecision of body composition measurement for small changes in fat mass. Causal relationships in this case can be interrogated by pair-feeding or other methods to control energy intake.

^b^Findings vary based on experimental protocol.

^c^Independence of energy intake evidenced by metabolic defect (e.g., altered substrate partitioning, reduced energy requirement during development of obesity).

Clearly, genetic factors influence human obesity risk, with BMI heritability estimated at 30% based on whole genome sequencing [115]. Only a small component of this heritability can be explained by known common variation at ~290 single-nucleotide polymorphisms and the physiological consequences of most of these polymorphisms remain unknown. In some cases (e.g., *MC4R)*, common variation near genes known to cause monogenic obesity illustrates the critical importance of the central nervous system [116]—although these do not exclude pathways consistent with the CIM (Table 2). Some implicated genes are expressed widely in the brain and others are ubiquitously expressed (e.g., *FTO*). Still others are more prominently expressed outside the brain (e.g. *MSX1*, *TMEM18*, *SEC16B*, *ADCY3*). Indeed, pathway analysis showed that genetic susceptibility to obesity can involve “insulin secretion/action, energy metabolism, lipid biology and adipogenesis” [117].

For polymorphisms cited by Hall et al. [9] as evidence against the CIM, alternative interpretations remain viable. Homozygous mutations in *ATGL*, for instance, resulting in defective lipolysis do not appear to increase risk for obesity. However, this mutation also impairs lipogenesis, resulting in not only less fat mobilization, but also less fat storage. As Schreiber et al. [118] conclude, “Interdependence of lipid catabolism and synthesis provides a rational explanation for the lack of obesity in ATGL-deficient mice and humans.” Whereas alleles of the *FTO* gene are associated with appetite or food intake, this observation provides no information regarding metabolic pathways or causal direction.

Thus, the genetics studies indicate pathways involving obesity that operate within and outside the brain; in many cases, these appear consistent with the CIM. Altogether, genetic expression data do not definitively differentiate between the two models, in view of the brain’s role in controlling both food intake and energy metabolism and the communication between the body and brain through neural, metabolic, and hormonal signals.

### Epidemiology

Although design limitations preclude a direct test of causal mechanisms in the EBM vs CIM with observational research, these studies can still be informative if interpreted with the necessary caution. Hall et al. [9] state that “evidence to suggest that carbohydrate intake explains between-country differences in body weight is nonexistent”, but these ecological comparisons are of little value for a variable like body weight. Countries with high carbohydrate intake, for instance, tend to be poor, with a substantial proportion of the population undernourished, malnourished, and engaged in subsistence agriculture. Moreover, Hall et al. disregard a long and rich history of observations linking the emergence of common chronic disorders, obesity among them, to population-wide nutrition transitions that typically include increased consumption of highly refined grains, sugar, and sugary beverages [119, 120]. In the USA, BMI increased most rapidly from 1970 to 2000, also concurrent with marked increases in consumption of refined grains, sugar, and total carbohydrate [121, 122]. These secular trends, though, may be confounded by changes in physical activity and other relevant behaviors.

Prospective cohort studies provide greater ability to control for confounding factors, notably including socioeconomic status, although residual confounding may remain. In addition, body weight and other measures of adiposity are especially susceptible to reverse causation (the tendency for people to change their diets as a result, rather than a cause, of weight gain or obesity). Furthermore, the typical prospective design comparing baseline diet with future weight change will not detect prior changes that have reached steady state by the time of the dietary assessment. In this situation, bias toward null associations may ensue; thus, the lack of consistent association involving GI and GL in cohort studies is difficult to interpret [123]. To better simulate an interventional study, the relationship of change in diet to change in weight over time can be examined. In such analyses, higher intakes of refined grains, potato products, and sugar-sweetened beverages—the main contributors to GL—were associated with greater weight gain in three large cohorts after extensive adjustment for potentially confounding dietary and lifestyle factors [124]. (Red and processed meats were also associated with greater weight gain in these studies.).

Hall et al. [9] conclude that the epidemiological data, “consistent with the EBM, suggest a variety of potential dietary drivers of excess calorie intake…” However, Mozaffarian [125] raises new questions about this conceptualization, at least as pertains to the current stage of the obesity epidemic in the USA. Based on nationally representative surveys, Mozaffarian notes that energy intake has plateaued or declined since 2000, and physical activity has increased moderately, even as rates of obesity continue to rise. (Because of disproportionate increases in waist circumference in women, obesity trends as assessed by BMI may underestimate the extent to which the epidemic has advanced since 2000 [126]). These trends, he argues, call for consideration of alternative causal explanations, including those involving metabolic dysfunction.

### Clinical trials

A recent meta-analysis of behavioral trials reported no difference in long-term weight loss among macronutrient-focused diets [127], as cited by Hall et al. [9], whereas other meta-analyses comparing low- vs. high-carbohydrate diets suggest a significant, if modest, advantage to the former [128–131]. However, interpretation of this evidence tends to conflate efficacy with behavioral implementation [132]. Most behavioral weight loss trials lack sufficient intervention intensity to obtain strong contrasts in macronutrient intakes between groups, and initial differences in weight loss between groups wane rapidly. Maintenance of dietary change can be difficult in the modern food environment, but this challenge is not insurmountable. With better knowledge of efficacy, more powerful behavioral and environmental interventions can be designed to facilitate long-term adherence. Among the few trials that employed intensive interventions (e.g., partial food provision), weight loss was greater on low- vs. high-GL diets for the duration of the protocols [133, 134].

The limitations of free-living trials can be, in principle, circumvented by metabolic ward trials that maintain strict control over adherence and confounding factors. However, due to cost and logistical challenges, these trials are usually short in duration, raising concern for unfounded inference involving chronic effects. The need for trials of at least several months duration was recognized by Hall [20], who observed that:

“even small differences in energy expenditure and macronutrient balance can theoretically lead to significant differences of body weight and composition if the diets are maintained over long periods. A 100 kcal/d difference in energy expenditure alone could lead to an initial body fat imbalance of about 10 g/d. Using current body composition methods, it would require a sustained period of about 100 days to detect such a difference in body fat. Nevertheless, this possibility requires further investigation.”

Furthermore, metabolic adaptations to macronutrient changes may require several weeks to months [135–141]. A recent meta-analysis reported higher total energy expenditure, with low heterogeneity, among studies ≥2.5 weeks duration comparing low- vs. high-carbohydrate diets [142]. No meaningful dietary effect was evident in studies <2.5 weeks, with substantial heterogeneity, reinforcing concerns about the value of short trials. The artificial setting of a metabolic ward may also affect eating behavior independently of underlying physiological mechanisms.

Hall et al. [9] interpret two 2-week inpatient trials as inconsistent with the CIM. In one of these trials [143], ad libitum energy intake was ~500 kcal/d greater on an “ultra-processed” vs. “unprocessed” diet [9]. However, this difference waned rapidly, with a slope of −25 kcal/d on the “ultra-processed” diet, suggesting the effect could extinguish after an additional 2 weeks. Furthermore, the initial difference in energy intake was fully attributable to the large difference in energy density, a factor that affects short-term, but not chronic, intake (see below). Related to this concern is the inability to distinguish crucial macronutrient mechanisms. Whereas the extent of food processing greatly affects digestion rate, hormonal response, and health impacts of high-carbohydrate foods, processing has lesser physiological significance for high-fat and high-protein foods (Table 3)—implying that the adverse effects of ultra-processed foods can be better explained by the CIM than by the EBM.

**Table 3:** Macronutrient-dependent effects of food processing.

Native food structure	Disrupted food structure	Major processing-dependent health effects
High-carbohydrate foods [230–234]		
Wheatberries	White bread	+
Oat groats	Instant oatmeal	+
Apple	Apple juice	+
High-fat foods		
Olives	Olive oil	–
Peanuts	Peanut butter	–
Avocado	Guacamole	–
Sesame seeds	Tahini	–
Cacao	Dark chocolate	–
Heavy cream	Whipped cream	–
High-protein foods		
Turkey	Ground turkey	–
Soybeans	Tofu	–
Boiled egg	Scrambled egg	–

The cellular structure of plants, including cellulose and soluble fibers, protects intrinsic carbohydrates from enzymatic digestion and diffusion to the gut wall. Extensive food processing disrupts this structure, resulting in acellular starches and sugars with markedly increased GL and adverse health effects. With inherently slower digestion rate, high-fat and high-protein foods are less affected by food processing. (Although sometimes designed with animal-sourced foods [144], a low-GL diet may be vegetarian or vegan, as most of these examples highlight.).

A similar pattern of effect attenuation, potentially related to metabolic adaptation and energy density, was observed in a second 2-week ward trial comparing low-fat vs. low-carbohydrate diets [144]. Pending definitive research, it seems prudent not to assume that these waning effects would stabilize and influence body weight over the long term.

### Drugs

A dominant role of insulin on adipocyte physiology, including lipogenesis and lipolysis, has been recognized for decades [145]. In patients with diabetes, insulin and drugs that increase insulin secretion or action on adipose tissue metabolism cause weight gain [146]. Some of these effects may involve other mechanisms compatible with EBMs, such as reduced glycosuria. However, the weight loss induced by drugs that lower secretion [147] suggests that the action of insulin on fat storage seen in rodents [103–109] occurs in humans. For instance, alpha-glucosidase inhibitors [148], which lower the glycemic response to carbohydrate, produce weight loss of ~1 kg, while also lowering HbA1c, in contrast to some other diabetes drugs (including insulin) that cause weight gain. Drugs that lower insulin secretion in people without diabetes also cause weight loss [147]. Furthermore, two new studies suggest that insulin suppresses adipose mitochondrial respiration in humans [149, 150].

Hall et al. [9] consider the effectiveness of GLP-1 receptor agonists for obesity as evidence against the CIM, because this incretin acutely potentiates glucose-stimulated insulin secretion. However, GLP-1 has other relevant biological actions, including reduced gastric emptying rate (which lowers glycemic response) [151]. In fact, GLP-1 receptor agonists chronically reduce measures of total insulin secretion [152, 153], although whether this effect is direct or indirect remains unclear. In any event, dietary GL strongly affects the incretin secretion profile and incretins have direct actions on adipocyte insulin sensitivity. For these reasons, GLP-1 lies on the central causal pathway in the CIM [8].

Regarding inhibition of lipolysis, Hall et al. [9] cite a study showing no effect of acipimox on weight in humans [154]. However, this nicotinic acid receptor agonist has biological actions that complicate interpretation of the trials. Acipimox increases counter-regulatory hormone secretion, promotes protein breakdown, and induces a compensatory increase in glucose oxidation [155]. Of note, inhibition of fatty acid oxidation with various agents stimulates food intake in experimental animals and humans [156–161].

To summarize evidence pertaining to the two models, the animal data demonstrate that excessive fat deposition can evidently be disassociated from energy intake, opposing a fundamental premise of the EBM. In animal models involving not only diet, but also brain pathways considered to mediate food intake, obesity can occur without increased food intake. However, the human data have major methodological limitations that have, so far, precluded a definitive test of the two models. To advance science, studies with adequate duration and complementary designs will be needed, including: (1) mechanistically oriented feeding studies capable of distinguishing transient from chronic macronutrient effects (≥1 month); (2) efficacy trials with adequate intervention intensity to produce meaningful long-term behavior change (≥1 year); and (3) longitudinal observational studies, ideally beginning in childhood, of the natural history of obesity (≥10 years).

## Clinical translation and public adoption

Both sides of this debate agree that fundamental changes in the food environment have driven the obesity pandemic. The new EBM’s focus on such a broad range of dietary factors offers few new actionable insights (ubiquitous, cheap, convenient, energy-dense, ultra-processed foods high in portion size, fat, and sugar, and low in protein and fiber). The implicit advice, to avoid junk foods, has been advocated for years [56, 162–166]. Of particular concern, causal relationships with chronic weight gain have not been demonstrated for the dietary factors targeted by Hall et al. [9] other than those that also involve CIM-related pathways (i.e., sugar, which is high in GL and fructose; fiber, which lowers the GI of co-ingested carbohydrates; and protein, which lowers the GI of co-ingested carbohydrates and stimulates glucagon secretion). The remaining EBM-specific dietary targets include:

- *Energy density*. Acute changes in energy density affect short-term intake. For example, Bell et al. [167] gave 18 women, in a cross-over design, diets differing in energy density but controlled for macronutrients. The women consumed the same volume of food during each condition, resulting in a 31% increase in energy density and a corresponding 31% increase in energy intake on the high- vs. low-energy-density conditions over 2 days. Hall et al. [9] cite several interventions and one observational analysis to suggest an important chronic effect. In one interventional study [168], 97 women with obesity were counseled to decrease fat intake alone or to decrease fat intake and increase low-energy-density fruits and vegetables. After 1 year, completers in the low-energy density group lost 1.5 kg more than those in the comparison group, but the effect related to greater loss of lean mass. The groups did not differ in total fat mass or waist circumference. In another interventional study [169], 200 adults were counseled to follow energy-restricted diets, with some instructed to consume varying amounts of low-energy-density soups vs. high-energy-density solid snacks. Here again, there was a modest difference in body weight at 1 year. However, participants in the snack group consumed exceedingly high-GL items (“crackers, baked potato chips, baked tortilla chips, bagel chips, and pretzels”); not surprisingly, carbohydrate consumption was greater in this group, precluding any relevant causal inference. Furthermore, in the largest and longest trial of this question (*n* = 2718), a significant difference in energy density between intervention groups was maintained for 4 years, with no effect on energy intake or body weight [170]. Regarding observational data on energy density [171], Bes-Rastrollo et al. [172] highlight major concerns about confounding and generalizability.
- *Dietary fat*. Assumptions about the role of energy density in obesity motivated, in large measure, the focus on reducing dietary fat in public health recommendations from the late twentieth century [173–177]. However, low-fat diets have not shown superiority for obesity-related outcomes [178–180], and some meta-analyses conclude inferiority vs. higher-fat diets for weight loss [128–130]. The USDA has virtually abandoned the public health campaign to reduce total dietary fat [181].
- *Food processing*. Food intake was greater in a 2-week trial with consumption of an “ultra-processed” vs. “unprocessed” diet [143]. However, this effect, a ∼20% increase, is attributable to the ∼85% increase in non-beverage energy density alone, based on the findings of Bell et al. [167]. A systematic review of observational data by Poti et al. [182] concludes, “It remains unclear whether associations [with obesity] can be attributed to processing itself or the nutrient content of ultra-processed foods.… and the potential for residual confounding was high.” As demonstrated in Table 3, macronutrient composition affects how disruption of native matrix and structure of a food alters health effects, suggesting that CIM mechanisms offer a better explanation for the associations of ultra-processed foods with obesity than those of the EBM.

Although the continuing increases in obesity prevalence might be attributable to lack of public adoption rather than any inherent deficiency of the EBM itself, the results of EBM-guided treatment throughout the last century suggest otherwise. In 1959, psychiatrist and obesity researcher Albert (“Mickey”) Stunkard with Mavis McLaren-Hume [183] conducted a 30-year literature review dating back to the original use of calorie counting for weight control in the 1920s. They concluded that the outcomes among reports were “remarkably similar and remarkably poor” and that these results “poor as they seem, are nevertheless [probably] better than those obtained by the average physician.” Explicitly addressing the notion of energy balance, the authors wrote:

“Many years ago detailed metabolic studies demonstrated that human beings do not defy the … law of thermodynamics and that excessive body fat results from an excess of caloric intake over caloric expenditure. This not unreasonable finding was thereupon enshrined as the dictum that ‘all obesity comes from overeating’… The physician’s job, it seemed, was simply to explain that semistarvation reduces fat stores, to prescribe a diet for this purpose, and to sit by. If the patient lost weight as predicted, this merely confirmed the comfortable feeling that treatment of obesity was really a pretty simple matter. However, if, as so often happened, the patient failed to lose weight, he was dismissed as uncooperative or chastized as gluttonous. It was the rare physician who entertained the possibility that failure to follow a regimen might in itself be a medical problem.”

In 1992, the National Institutes of Health sponsored a Consensus Development Conference on Methods for Voluntary Weight Loss and Control, including many of the leading experts in obesity. At that time, dietary fat restriction was considered “The best means of achieving a healthy weight … preferred because it is easier to eat fewer calories without having to eat small portions” [184], a view frequently espoused in contemporary academic reviews [173, 174]. However, the Consensus Conference found little evidence that obesity treatment achieved much better outcomes that those reviewed by Stunkard and McLaren-Hume [183]. Conference proceedings concluded that “participants who remain in weight loss programs usually lose approximately 10% of their weight…. [much] of the weight is regained within 1 year, and almost all is regained within 5 years” [185]. Moreover, the analysis of Mozaffarian [125] provides quantitative evidence that, in recent decades, Americans have adhered to the fundamental “eat less” recommendation of the EBM, at least on a population basis – even as obesity rates continue to increase.

Axiomatically, disease treatment focused on causal drivers (upstream along the mechanistic pathway) should be more effective, and more sustainable for the patient, than those targeting downstream consequences and manifestations. If fever were, by analogy, considered a disorder of “heat balance,” one might rationally prescribe a cold shower to reduce body temperature. This treatment would work temporarily (if one could convince a febrile patient to try it), but the body would compensate for the heat loss by severe shivering and blood vessel constriction. Once the patient got out of the cold shower, the fever would return. Antipyretics work more effectively, and more pleasantly for the patient, by addressing the biological driver of heat accumulation. Similarly, if obesity results from a disorder of fuel partitioning, then measures to treat that problem (e.g., by reducing the insulin-to-glucagon ratio) would achieve better adherence than calorie restriction, because the patient would experience less hunger and a lesser reduction in energy expenditure with weight loss.

## Muddling paradigm clash

Maintaining the contrast between these competing models is critical to clarify thinking, inform a research agenda, and identify effective means of prevention and treatment. Hall et al. [9] muddle this contrast by relegating the CIM to “a special case” of the EBM. This claim belies the most fundamental possible differences among models: causal direction and mechanisms of causality (Fig. 1). To subsume the CIM in this way requires construing the EBM so broadly as to make it unfalsifiable, and consequently useless as a scientific hypothesis. As Karl Popper reportedly said, “a theory that explains everything, explains nothing.”

Hall et al. [9] also claim that the CIM has abandoned fundamental precepts, referring to prior “adipocentric” formulations said to consider only the actions of insulin in adipose tissue. However, this characterization was not made by CIM proponents and offers a false distinction. The control of adipose tissue biology by multiple hormonal, autonomic and other influences has been recognized for decades [27]. Indeed, the physiological actions of high-GL and high-sugar diets have long been conceptualized as involving integrated relationships among multiple organs beyond adipose tissue and numerous hormones beyond insulin [6, 29].

This concern about CIM revision contrasts with their acknowledgment that “development of the EBM [still] requires elucidation of the factors in the dynamic food environment that are most responsible for instigating obesity [and] the mechanisms by which these factors alter the brain circuits controlling food intake” [9]. Indeed, dietary targets of EBM-based recommendations have changed from calorie counting in the early twentieth century [186] to an overarching focus on dietary fat restriction in the late twentieth century [173–177, 187], to the notion that all calories are alike [2, 18, 19], to the new formulation [9], subtitled “beyond calories in, calories out,” that now blames a host of modern dietary factors. For scientific models to remain relevant, they must grow as knowledge accrues.

Even as Hall et al. [9] criticize the provenance of the CIM, their EBM has major deficiencies, including:

- *Lack of explicit testable hypotheses*. How will key steps along the causal pathway be interrogated? What studies will differentiate the proposed causal pathway (overeating drives chronic weight gain) from the contrasting hypothesis in the CIM? When humans or animals are experimentally overfed, they gain weight initially. But changes in hunger and energy expenditure oppose ongoing weight change; after the force-feeding ends, individuals characteristically undereat until body weight returns to baseline [188–193]. In other words, the excess energy “pushed” into adipose tissue doesn’t stay “put” [4], yet excess adipose mass accumulated over time on habitual diets remains remarkably stable.
- *Tautologies*. While arguing that opponents of the EBM confuse physics with pathophysiology, Hall et al. assert that, “the EBM incorporates physiological mechanisms underlying energy partitioning … such that overall energy imbalances are primarily reflected as fat imbalances regardless of the composition of the diet.” They also assert that “whole-body fat imbalances end up primarily reflected as changes in adipose tissue fat storage.” In so doing, they propagate this confusion. As considered above, the law of energy conservation holds that a change in energy balance must coexist with a commensurate change in fat and adipose tissue mass (the body’s main energy storage biomolecule and depot, respectively). These tautologies provide no mechanistic insight.
- *Paucity of mechanisms involving key model components*. How does the new EBM explain the rapid population-level increase in weight, and large variations within individuals over time? Physiologically regulated variables (e.g., body temperature, serum sodium) are characterized by stability except under extreme conditions. What studies would distinguish the putative mediators (e.g., reward, hedonic influences) from those in the CIM (hormonal response to macronutrient composition)? Moreover, if pleasure-related responses to tasty foods cause chronic overconsumption, why has it been so difficult to demonstrate an independent effect of palatability on obesity [194–201]?
- *Disregard of well-established metabolic mechanisms*. For individuals with obesity, energy restriction elicits hallmarks of the starvation response (including reduced energy expenditure) long before body fat stores reach a normal level. How do the hedonic and reward aspects of palatable food trigger metabolic responses?
- *Difficulty accounting for the natural history of obesity*. Most forms of obesity develop over many years, associated with a positive energy balance of ∼10 to 20 kcal/d (the energy content in 1 teaspoon of sugar). The secular increase in energy intake from 1970 to the present in the U.S. is ∼200 kcal/d (12 oz grape juice) [122, 125, 202]. Considering the psychosocial and other burdens of excessive weight, why do so few people successfully compensate by conscious control for these small daily effects? After all, adults routinely resist pleasurable temptations (e.g., sex, drugs) that also recruit subconscious drives?
- *Reliance on assumptions that do not differentiate among models*. The new EBM interprets evidence that the brain controls body weight as supporting a causal role of overeating in obesity. As considered above, the brain also influences virtually all aspects of energy metabolism and adipocyte biology.

## Conclusions

For intractable public health problems, the purpose of scientific models is to guide the design of informative research and, by helping to elucidate causal mechanisms, suggest effective approaches to prevention or treatment. The new EBM does neither. At a minimum, future formulations should (1) specify testable, mechanistically oriented predictions that examine the causal pathway; (2) explain why the increased population-level BMI is defended by metabolic responses; and (3) demonstrate how calorie-independent effects of diet suggested by clinical research and demonstrated by animal models can be integrated in this model.

The EBM and its precursors have dominated thinking for nearly a century [7]—influencing scientific design, interpretation of experimental findings, public health guidelines, and clinical treatment—largely to the exclusion of other views. For instance, the NIH has sponsored numerous multi-center trials of low-fat diets for obesity-related outcomes [178–180] (all with negative primary outcomes), but nothing comparable for low-GL diets. With the inability of conventional strategies to stem the rising toll of obesity-related disease, new causal models should be studied, not suppressed by hyperbolic claims of having disproven them [2, 9, 18, 19, 57, 58, 203–205].

Admittedly, debate on complicated scientific questions may polarize, with a tendency for both sides to cite selectively from inconclusive evidence. This problem is exacerbated by difficulties in studying the small daily effects that characterize the natural history of obesity. In the interests of scientific advancement and public health, all sides of this debate should work together to formulate mutually acceptable versions of competing models and design unbiased studies that would put them to a rigorous test. A constructive paradigm clash may be facilitated with the recognition that evidence for one model in certain experimental settings does not invalidate the other model in all settings, and that obesity pathogenesis in humans may entail elements of both.

Finally, we would emphasize that this paradigm clash should not delay public health action. Refined grains and added sugars comprise about one-third of energy intake in the US and Europe. Both models target these highly processed carbohydrates—albeit for different reasons—as major drivers of weight gain. Regardless of how this debate may evolve, common ground now exists on the need to replace these products with minimally processed carbohydrates or healthful fats in the prevention and treatment of obesity.

## Data Availability

No original data were used in this review.
